# Internal Jugular/Subclavian Venous Access In Electrophysiology Study And Ablation

**Published:** 2009-07-01

**Authors:** Shomu Bohora, Jaganmohan Tharakan

**Affiliations:** 1 Consultant, Baroda Heart Institute and Research Centre, Vadodara, India; 2 Professor and Head of Department of Cardiology, Sree Chitra Tirunal Institute for Medical Sciences and Technology, Trivandrum, India

**Keywords:** Electrophysiology study, Venous access, Internal jugular vein, subclavian vein

## Abstract

Multiple venous accesses are required for catheter placement during electrophysiology study and ablation. Internal jugular/subclavian venous access, though restricted nowadays, can be important in difficult situations.

## Introduction

Evolution of mankind and science is a continuous process. The best and the fittest survive, while the others perish over a period of time. Charles Darwin's theory of evolution of mankind can also be considered for scientific evolution. Cardiology has advanced leaps and bounds in our current understanding, for diagnostics and treatment of disease and also has made advanced procedures available to a common man. The evolution in healthcare infrastructure has led to better disease diagnosis and treatment and overall improved patient care. Older and outdated methods of diagnosis and treatment have fast perished in the fast evolving healthcare sector.

Electrophysiology (EP), as a subspecialty in cardiology, has also witnessed a major change in diagnostic and treatment capabilities [1-3]. Not only the time taken for the procedure is reducing, but the current advancements have made the procedure more safe and dependable. Newer hardware and tools like 3 D anatomical mapping have evolved, making it easier to diagnose and treat even complex arrhythmia [1-3]. Routine EP study requires the placement of multiple catheters, traditionally 4 catheters, for appropriate recordings and diagnostics. This requires multiple venous accesses to be taken. Femoral veins offer the best access sites to position the EP catheters within the cardiac chambers. Internal Jugular vein (IJV) or subclavian venous access have been used traditionally for placement of fixed curve decapolar catheters for coronary sinus recordings, due to easier cannulation of coronary sinus from superior aspect. In this article we would review the importance of superior venous access in electrophysiology studies and its changing scenario.

## Anatomical and hardware related issues for obtaining appropriate recordings and selection of venous access sites

 Routinely, one recording catheter is placed each in the right atrium, right ventricle, tricuspid annulus in the His bundle region and the coronary sinus during an EP study.  All these regions are accessed from the right side of the heart and hence only venous access is required during a standard EP study. Arterial access is used for mapping the left ventricle, aorta and the mitral annulus and for pressure monitoring. Depending on the site of ablation, a venous or arterial access for ablation catheter in addition is required during ablation. Hence on a routine 5 catheters are required during a routine EP study and ablation for which 4 or 5 venous access need to be taken. However after many years in practice and evolution of diagnostic maneuvers, arrhythmias can be diagnosed and treated reliably with lesser number of catheters [4,5].

Most of the routinely used EP catheters are either fixed curve or deflectable distally. They are more rigid when compared to the catheters used in interventional cardiology. Most of the catheters are non-luminal catheters and hence cannot be tracked over a wire in case of vessel being tortuous. Hence venous accesses which offer the least tortuous course offer the best access sites. Access from the femoral veins, right internal jugular vein and the left subclavian vein offer the course of least resistance because the angulations further in their course are gentle. Also since these veins are large in adults and can accommodate larger and multiple sheaths, multiple and large size catheters, which are routinely used during EP study, can be passed through a single vein easily in adults.

Routinely the venous access taken during EP study is the femoral vein through which 2-3 catheters can be placed through each vein. In addition when a fixed curve decapolar catheter is used for coronary sinus recordings, a right IJV or a left subclavian venous access is routinely being used, which offers easy cannulation of the coronary sinus. With the availability of deflectable quadripolar/decapolar catheters, coronary sinus cannulation can easily be done from the femoral venous route and there exists hardly a need for either IJV or subclavian venous access in a routine EP study and RFA. Occasionally femoral veins in their course to the inferior vena cava (IVC) can be tortuous, making passage of EP catheters difficult, but the problem can be circumvented easily by using long sheaths through the femoral veins into the IVC.

## Conditions in which femoral access route may be difficult or not feasible and hence superior venous access is required

Congenital anatomical anomalies of the femoral veins, iliac veins or the IVC in the form of smaller vessel size, bifurcating veins, excessive bends and tortuous course, interruption or agenesis may lead to impossible or difficult catheter placements from the femoral venous route. Partial obstruction of veins by either, membrane, stenosis or thrombosis which may have been asymptomatic may also present difficulties. Iatrogenic blocks, like IVC filters for preventing pulmonary thromboembolism, would possibly contraindicate femoral venous access in certain patients. In such conditions most or all of the catheters may need to be placed from the superior route and has been described before in various reports [6-14]. In this issue of the journal, in case report by Karthigesan et al [15], of a patient having left sided IVC with hemiazygous continuation, placement of catheters through the right IJV was done due to inability to position catheters from the femoral access route. Also ablation was done from the IJV access. In the case report by Jorg et al [16], for ablation of atrial flutter, all catheters were placed from the superior route because of the presence of an IVC filter.

## Conditions in which target site for recording or ablation is better accessed from the superior venous route

A superior route of access for recording signals from the coronary sinus is easier as catheter cannulation of the coronary sinus ostium is easier and even fixed curve decapolar catheters can easily be introduced within the coronary sinus from either the right IJV or the left subclavian vein. However, with the availability of deflectable decapolar and quadripolar catheters, coronary sinus cannulation and subsequent catheter placement and recordings can easily be obtained through the femoral venous access. Hence for routine diagnostic EP procedures when deflectable catheters are used, only femoral access needs to be taken in most cases.

Ablations for true posteroseptal pathways, which may be epicardial, and have to be ablated from within the coronary sinus, a superior route may offer better success and easy maneuverability [17]. In cases of failed ablation from the femoral route, a superior route of access of ablation catheter for ablation within the coronary sinus or the middle cardiac vein has been successful primarily because of the reach and apposition to target tissue of ablation is much better from the superior route. Sometimes right free wall accessory pathway ablation when required to ablate from under the tricuspid valve leaflets, right anterior  and para-hisian accessory pathway ablation may require approach from the superior route, so as to obtain a stable catheter position. However with the availability of long sheaths of different curves to achieve better catheter stability and ablation catheter with bi-directional distal tip, a superior route of ablation may hardly be required for accessory pathway ablation.

Pediatric EP studies have become much more common than before and are been done in very young age groups also. A major source of concern in pediatric procedure is the limitation of venous access because of the small sized vessels. Even with availability of smaller sized catheters, one or two catheters only can be passed from each of the femoral veins. Also coronary sinus cannulation is much more difficult from the femoral access route. Therefore, especially in pediatric age group, IJV and subclavian veins access for catheter placement is much more of a necessity when compared to the adult age group [18-20]. Esophageal pacing in substitute for atrial catheter can be used to limit the number of venous access sites in children [21].

## Problems and disadvantages of a superior venous access

In the era of modern medicine, patient comfort is as important as a safe and successful procedure. Patient comfort during an EP study is best with femoral only access. Manipulation in the neck and chest region is associated with more discomfort during the procedure especially with the prolonged time a patient is not allowed to move the neck or the hands when a superior access is taken. Also superior venous accesses are more likely to have venous access related minor and major complications as compared to a femoral venous access. Access scar marks from the superior route may not be acceptable to some patients.

Not only does the patients feel comfortable with a femoral venous access, the operator too is comfortable to move the catheters from down below rather than from the patients head end. With the usual training, the procedural skills of an operator for catheter manipulation from the femoral route, is much more precise and easier. Catheter manipulation from the superior route, except for cannulation of coronary sinus would require extra effort and time. The expected movements of catheters from the superior route may sometimes be imprecise due to non familiarity of position and anatomy, which would translate in more risk for causing complications, especially during ablation near the His bundle region. Innovative maneuvers that achieve better catheter stability are required, as has been described by Jorg et al [16] in their case report of ablation of atrial flutter. Radiation exposure for the operator is higher when working from the head end of the patient and with the increased fluoroscopy times; it translates into an increase of total radiation exposure to both the patient and the operator.

## Summary

Internal jugular and subclavian venous access has been fast replaced by only femoral venous access during routine electrophysiology procedures due to availability of operator friendly hardware and greater patient comfort. However for placing catheters and ablation within the coronary sinus, pediatric procedures and situations in which obtaining femoral access for catheter placements is difficult, access through the superior route comes to the rescue and offers a chance for diagnosing and treating the arrhythmia successfully. Rather than extinction, access through the internal jugular and the subclavian veins would continue to enjoy the part as a rescuer for the times to come.
